# Acute coronary syndrome in young Sub-Saharan Africans: A prospective study of 21 cases

**DOI:** 10.1186/1471-2261-13-118

**Published:** 2013-12-14

**Authors:** Moustapha Sarr, Djibril Mari Ba, Mouhamadou Bamba Ndiaye, Malick Bodian, Modou Jobe, Adama Kane, Maboury Diao, Alassane Mbaye, Mouhamadoul Mounir Dia, Soulemane Pessinaba, Abdoul Kane, Serigne Abdou Ba

**Affiliations:** 1 Service de Cardiologie, CHU Aristide Le Dantec University Hospital, PO Box 6633, Dakar étoile, Avenue Pasteur, Dakar, Senegal; 2 Service de Cardiologie, Hôpital Général de Grand Yoff, Dakar, Senegal

**Keywords:** Acute coronary syndrome, Young Sub-Saharan African, Dakar

## Abstract

**Background:**

Coronary heart disease remains the leading cause of death in developed countries. In Africa, the disease continues to rise with varying rates of progression in different countries. At present, there is little available work on its juvenile forms. The objective of this work was to study the epidemiological, clinical and evolutionary aspects of acute coronary syndrome in young Sub-Saharan Africans.

**Methods:**

This was a prospective multicenter study done at the different departments of cardiology in Dakar. We included all patients of age 40 years and below, and who were admitted for acute coronary syndrome between January 1^st^, 2005 and July 31^st^, 2007. We collected and analyzed the epidemiological, clinical, paraclinical and evolutionary data of the patients.

**Results:**

Hospital prevalence of acute coronary syndrome in young people was 0.45% (21/4627) which represented 6.8% of all cases of acute coronary syndrome admitted during the same period. There was a strong male predominance with a sex-ratio (M:F) of 6. The mean age of patients was 34 ± 1.9 years (range of 24 and 40 years). The main risk factor was smoking, found in 52.4% of cases and the most common presenting symptom was chest pain found in 95.2% of patients. The average time delay before medical care was 14.5 hours. Diagnosis of ST- elevation myocardial infarction in 85.7% of patients and non-ST-elevation myocardial infarction in 14.3% was made by the combination electrocardiographic features and troponin assay. Echocardiography found a decreased left ventricular systolic function in 37.5% of the patients and intraventricular thrombus in 20% of them. Thrombolysis using streptokinase was done in 44.4% of the patients with ST- elevation myocardial infarction. Hospital mortality was 14.3%.

**Conclusion:**

Acute coronary syndrome is present in young Sub-Saharan Africans. The main risk factor found was smoking.

## Background

Coronary heart disease remains the leading cause of death in developed countries. It is usually seen from the fifth to the seventh decades of life, but some cases in young people have been reported [1,2]. In Africa, while the work of the first European doctors that arrived on the continent claimed the low occurrence or even absence of atherosclerosis and its clinical manifestations, different schools of cardiology have shown not only their emergence, but especially their increase although at different rates in different countries [3,4]. On the other hand, few studies have been published on young people (5).

The objective of this work was to study the epidemiological, as well as the clinical and evolutionary peculiarities of acute coronary syndrome (ACS) in the young Sub-Saharan Africans of age 40 years and below.

## Methods

This was a prospective multicenter study conducted at the respective cardiology departments of Aristide Le Dantec University Hospital, Grand Yoff General Hospital and Principal Hospital of Dakar over a period of 31 months (January 1^st^ 2005 to July 31^st^ 2007), in Dakar, Senegal.

All patients with an age of 40 years and below, admitted for acute coronary syndrome on the basis of anginal pain at rest, suggestive electrocardiographic changes and elevated troponin I levels, were included. Patients with more than 40 years of age, those with stable angina, and those with semi-recent or sequel of coronary syndrome were excluded from the study.

We studied data on age, gender, past history including history of diabetes, hypertension, smoking, alcoholism, sedentarism (less than 30 minutes or more of moderate-intensity physical activity on most days of the week), obesity; family history of coronary heart disease at a young age (before 55 years in men and 65 years in women), use of estrogen-progestin contraceptives, stable angina and stress.

We sought the presence of chest pain, dyspnea and gastrointestinal symptoms.

We also noted the time delay before admission, the management given, and the vital parameters (blood pressure, heart rate, respiratory rate, temperature, body mass index).

All patients had a complete physical examination and a laboratory assessment. Troponin I assay was done using Architect STAT chemiluminescent microparticle immunoassay (Abbot). The other tests included blood glucose level on admission, total cholesterol and its fractions, and triglycerides. On the ECG, we looked for subepicardial or subendocardial lesion, subepicardial or subendocardial ischemia, abnormal Q waves, rhythm and conduction abnormalities. We have also looked for signs of venous stasis on the chest x-ray and evaluated using Doppler echocardiography (which was performed during the first 24 hours of admission), the left ventricle wall motion, left ventricular ejection fraction using Simpson's biplanar method, and the presence of intracavitary thrombus. Treatment modalities were evaluated as well as evolution during hospitalization.

The study protocol was approved by the Ethics Committee of the Ministry of Health and Social Welfare, Senegal (Comité d’éthique du Ministère de la Santé et l’Action Sociale). A signed consent form was obtained from each of the study participants.

The studied parameters were entered into an electronic questionnaire using Epi Info version 6.0 of the World Health Organization. Data analysis was performed using SPSS 15.0 (Statistical Package for Social Sciences). Quantitative data were expressed as mean ± standard deviation.

## Results

Hospital prevalence of acute coronary syndrome in young patients was 0.45% (21/4627), meaning 6.8% (21/309) of patients admitted for acute coronary syndrome during the same period. Eighteen patients (85.7%) were males and three patients (14.3%) were females, giving a ratio of 6:1. The mean age of patients was 34 ± 1.9 years with a range of 24 and 40 years. In women, the mean age was 37 years and among men it was 34 years.

The risk factors in our patients (Table 1) were dominated by active smoking found in 11 patients (52.4%). The average number of pack-years was 8.10 ± 2.3 and ranged from 1 to 17 pack-years.

**Table 1:** Risk factors found in the study population

**Risk factors**	**Number**	**%**
Tobacco smoking	11	52.4
Stress	9	42.9
Sedentarism	3	14.3
Hypertension	3	14.3
Hypercholesterolemia	2	9.5
Heredity	2	9.5
Diabetes	1	4.8

Five patients (23.8%) had no risk factors, seven patients (33.3%) had one risk factor and the remaining patients (42.9%) had more than one.

Chest pain was present in 20 patients (95.2%). In 65% of cases, it was typical anginal pain and inaugural in 70% of cases. The average time delay before medical care was given was 14.5 hours, with extremes of 03 and 48 hours.

On admission, blood pressure was greater than or equal to 140 and/or 90 mmHg in three patients (14.3%). Average body mass index was 23.5 ± 1.5 kg / m^2^. No patient was found to be obese.

Systemic examination was strictly normal in 18 patients (95.2%). Three patients however showed signs of left ventricular failure (Killip II).

Mean troponin I level found was 29 ± 40.9 ng/ml (0.01 to 401.6 ng/ ml). Two patients had levels below 0.05 ng/ml. Average blood glucose level was 1.2 ± 0.4 g/l (0.6 to 3.83 g/l). It was found to be 1.24 g/l in 4 patients (21%). Mean total cholesterol was 1.4 ± 0.2 g/l (0.85 to 2.58 g/l). Hypercholesterolaemia was present in two patients, LDL cholesterol greater than 1.6 g/l in one patient and HDL cholesterol less than 0.4 g/l in seven patients (41.2%). Two patients had a triglyceride level greater than 1.5 g/l.

Electrocardiogram revealed an acute coronary syndrome with persistent ST-elevation in 18 patients (85.7%) and non ST-elevation acute coronary syndrome in three patients (14.3%).

Topographically, the anterior and inferior territories were the most represented and found respectively in 14 (66, 7%) and 7 patients (33.3%). An extension to the right ventricle was observed in one patient. We also noted two cases of ventricular tachycardia and complete atrioventricular block in one patient.

Chest radiography showed a cardiomegaly in four patients (21%). Doppler echocardiography revealed impaired segmental kinetics in 12 patients (60%). The mean ventricular ejection fraction was 52.2 ± 8.5% (20-80%). Systolic dysfunction was found in six patients and left ventricular thrombus in four.

Concerning treatment, thrombolysis using streptokinase was performed in eight patients, accounting for 44.4% of patients with ST-elevation.

Low molecular weight heparin was used in 19 patients (90.5%), aspirin in 20 patients (95.2%), clopidogrel in 7 patients (33.3%) beta blockers in 17 patients (81%), angiotensin converting enzyme inhibitors in 17 patients (81%), statins in 14 patients (66.7%) and analgesics in 13 patients (62%).

The evolution during hospitalization after a mean hospital stay of 18.7 ± 4.5 days (1–37 days) was favorable in 18 patients (85.7%). Three deaths (14.3%) were recorded, two of which were due to sudden death from ventricular fibrillation and one due to cardiogenic shock.

## Discussion

In our study, the prevalence of acute coronary syndrome before age 40 was 0.45% with an incidence of 6.8% on all patients with coronary heart disease hospitalized during the same period.

Data on the incidence of acute coronary syndrome in young people are rare in Africa [5,6]. However, Wade found a prevalence of 0.03% and an incidence of 5.7% [7].

This incidence of 6.8% is bringing our numbers in the range of the European and American literature (4-10%) [8-12] and could be an evidence of a real epidemiological transition.

The mean age of our patients was 34 years. Tricot [13], and Dolder [8] found, respectively, a mean age of 36 years and 35.4 years. However in India the youngest age reported for acute coronary syndrome was in a 14 year-old [14].

Our study confirms male predominance as has been emphasized in previous works [2,7,9,10,14].

Smoking was found to be the main risk factor and is often found in the occurrence of coronary events in young patients [15]. Indeed, most works on the occurrence of coronary syndrome in young patients have a disease pattern dominated by monotonous smoking [9,16,17].

This is reflected in our work where smoking is found in 52.4% of cases. This rate seems more important in European series, where it has been reported to be up to 93% [13]. The use of hard drugs, as was the case with one of our patients, may be associated with the occurrence of a coronary event [18,19].

The delay in our work (14.5 hours) is close to what is found in some Africans like in Tunisia [20].This period is shorter in the European series [21,22].

Clinically, acute coronary syndrome in young patients does not appear much different from that of the elderly. This has been emphasized by several authors [9,14,23,24]. The picture of acute coronary syndrome is dominated by pain in both the young as well as in the elderly.

Elevation of troponin levels form part of the definition of acute coronary syndrome [25]. This high rate of troponin assay in our work translates into a better integration of this biological parameter in the management of acute coronary syndrome. Concerning cardiomegaly found in 4 patients on chest X-ray at admission, no conclusion could be drawn as data on the existence cardiomyopathies or other heart disease could be established.

In our study we found using Doppler echocardiography an impaired left ventricular systolic function in 37.5% of cases versus 63.3% found by Renambot J et al. However, the mean age of patients in this series was 47.1 years ± 4 [26]. Left intraventricular thrombus in the apical region was found in 20% of our patients. These results confirm data from the literature [27,28]. Segmental abnormality observed in only 60% of the cases might be primarily the effect of thrombolytic therapy. Also the degree of myocardial damage may not have been severe enough to lead segmental abnormality.

The impact of thrombolytic therapy on mortality with a time-dependent effect has been reported by several studies [29-31]. It is now clear that thrombolysis can significantly reduce mortality of patients with acute coronary syndrome. In our work, steptokinase being the only available thrombolytic in our hospitals, was used in 44.4% of patients admitted with an ST-elevation acute coronary syndrome. The reasons for the low use of thrombolytics in this study were due to many factors including low awareness level of the population about acute coronary syndrome, poor transport and communication network to reach referral centers, lack of available electrocardiography in health centers which are located close to patients, delayed referral from health centers of suspected cases and inability to meet the high cost of thrombolytic treatment. The favorable outcome in young adults, as seen in our work (85.7%) seems higher than that observed in the elderly.

Heart failure is a common and serious complication of acute myocardial infarction. It was present in our work in 14.3% of patients. This was found in 18.2% and 9.2% respectively in the series of Al-Khadra and Kanitz [14,24]. A relationship between age and heart failure has been reported by Magid with a greater frequency of occurrence in the elderly compared to young subjects [32].

The ventricular arrhythmias are responsible for 30-40% of deaths occurring during the first 12 hours of a myocardial infarction. In our work, ventricular fibrillation was responsible for two deaths during the acute phase.

## Conclusion

The occurrence of acute coronary syndrome is a reality in young Sub-Saharan Africans. The main risk factor is smoking. Complications are not rare and the mortality remains high.

## Competing interests

The authors declare that they have no competing interests

## Authors’ contributions

MS, DMB, MBN and SAB designed the study protocol, participated in the data collection and contributed in analyzing the data and writing of the draft manuscript. MB, MJ, AdK and MD participated in data analysis and critically revising the manuscript for important intellectual content. AM, MMD, SP and AK participated in study design and in data analysis. All authors have read and approved the final version of the manuscript.

## Pre-publication history

The pre-publication history for this paper can be accessed here:

http://www.biomedcentral.com/1471-2261/13/118/prepub
